# Outcomes of a Comparison Study into a Group-Based Infant Parenting Programme

**DOI:** 10.1007/s10826-016-0489-3

**Published:** 2016-07-15

**Authors:** Catrin Hedd Jones, Mihela Erjavec, Simon Viktor, Judy Hutchings

**Affiliations:** 1 School of Healthcare Sciences, Bangor University, Bangor, Gwynedd Wales, UK; 2 School of Psychology, Bangor University, Bangor, Gwynedd Wales, UK; 3 Centre for Evidence Based Early Intervention, School of Psychology, Bangor University, Bangor, Gwynedd Wales, UK

**Keywords:** Positive parenting, Mother, Sensitivity, Infant

## Abstract

This paper reports on a quantitative evaluation of a group-based programme designed to promote parent-infant attachment and child development. Whilst group-based parenting programmes are recommended for treating and preventing conduct disorder in older children, there is, as yet, little evidence as to whether they have a positive effect on very young children and their carers’. Recent UK Government initiatives to support families and improve parenting skills in the first 2 years of children’s lives have increased the demand for the delivery and evaluation of community-based programmes. Eighty mother–child dyads were recruited from nine areas to intervention (n = 54) and control condition (n = 26). Baseline measures were collected in the children’s home when the infants were on average 3-months-old, and follow-up measures were collected 6 months post-baseline (N = 63). Mothers’ positive play behaviours were independently coded from video recordings taken in the home. Other measures included self-reported maternal confidence and mental well-being, assessed infant development and home environment. Socio-demographic data was collected once at baseline. After controlling for baseline scores, control mothers were observed to be significantly less sensitive during play with their baby at the 6 months follow-up with a significant increase in confidence. No differences were found between the groups on the other measures. This paper provides limited evidence for the effectiveness of the Incredible Years Parents and Babies group-based programme delivered in the first year of life. Further evaluation, particularly with parents at increased risk of poorer outcomes is needed to confirm and extend these results.

## Introduction

Parents are the main source of influence on their children’s development. The quality of interactions and continuity of response by a significant caregiver establishes a pattern of expectation in the infant. This supports the development of an ‘internal working model’ which can affect attachment security and future relationships (Ainsworth 1985; Bowlby 1969/1997; Guajardo et al. 2009; Meins et al. 1998; Rutter et al. 1998). Children who develop a secure attachment are more likely to be rated by teachers as independent explorers, demonstrate a strong sense of selfhood, adopt a flexible approach in peer interactions at pre-school and achieve better academic outcomes (Meins 1997; Pearson et al. 2011; Sroufre et al. 1983; Turner 1991).

Positive Parenting is defined as “the continual relationship of a parent(s) and a child or children that includes caring, teaching, leading, communicating, and providing for the needs of a child consistently and unconditionally”(Seay et al. 2014, p. 207). This definition reflects the diversity of skills required from parents. A positive relationship between parents and their children and the quality of children’s early environment can have long-term effects on their cognitive and behavioural development (Shonkoff 2011). Positive parenting can also support the development of infants’ coping mechanisms and decrease the risk of the externalising behaviour problems developing in later childhood (Boeldt et al. 2011).

Mothers who commented more on their 6-month-old infant’s internal mental states were observed to have infants who were classified as securely attached at 12-months of age. Increased parental awareness of their child’s internal mental state encourages the infant’s sense of security and fosters early social and emotional development (De Wolff and Van IJzendoorn 1997; Meins et al. 1998, Meins et al. 2001). Lack of appropriate communication has been shown to affect infants as young as 2-months of age. Experiments by Tronick and Cohn (1989) demonstrated that mother’s presenting a ‘still’ expressionless face to their infant initially provoked attempts by the infants to attract their mother’s attention; however, infants of depressed mothers, accustomed to lower levels of visual interaction, did not protest at the lack of interaction (Tronick and Gianino 1986). Infants who experience negative parenting or lack of stimulation become withdrawn and the resulting cyclical process of less rewarding interaction between infant and parent can be difficult to resolve, with increased risks of children developing conduct disorders (Lorber and Egeland 2011). Lower levels of adult speech and activity during interactions with 1-year-old infants significantly predict a diagnosis of child psychiatric disorder at 7 years of age (Marwick et al. 2013). The style of parenting can also impact on children with negative, controlling mothers more likely to have children who show increased problem behaviours in preschool (Spieker et al. 1999).

A study comparing the cognitive development (the Millennium Cohort Survey, N = 12,644) of children assessed from birth to 5 years of age reported an 11.1-month gap between low and middle-income children’s vocabulary test scores at 5 years of age (Waldfogel and Washbrook 2010). Multivariate analysis of the factors contributing to the gap showed parenting and the quality of the home environment to be the most important factors affecting the children’s scores. Parenting programmes have been shown to be effective in supporting parents’ mental well-being and confidence and this can have a long-term beneficial influence on the children’s social, economic and health outcomes (Bywater et al. 2009; Olds et al. 1998). Mothers identified at increased risk of poor parenting skills who received home visiting and group support for their child’s first year were more responsive to their infant’s communication and their children were more securely attached, more task-orientated and autonomous than the children of mothers who did not receive the support (Heinicke et al. 1999).

The well-being of parents and children are naturally intertwined and the transition into caring for their new infant could be an effective window of opportunity to support parents and influence positive parenting practice. O’Connell et al. (2015) described positive parenting as an “effective, yet underused, lever of paediatric outcomes” (p. 286). The World Health Organization (2016) defines mental health as a state of well-being in “which every individual realizes his or her own potential, can cope with the normal stresses of life, can work productively and fruitfully, and is able to make a contribution to her or his community”. The WHO’s report on Early Child Development (2007) stated that “What children experience during the early years sets a critical foundation for their entire lifecourse”. Recent cross-party UK political support for families in the first 2 years of life (Allen 2011; Commons 2015) resulted in debates in both the House of Commons and Lords supporting the ‘1001 Critical days’ manifesto and its vision that parents need to feel confident to raise their children in a loving and supportive environment. This manifesto represents a cultural shift towards early intervention to prevent problems from developing. Offering early support to parents is now viewed as a cost-effective investment to ensure all children, irrespective of their environment, get the best possible start in life (Allen 2011; Hall and Elliman 2006). A recent cost-benefit analysis estimated that by identifying 5-year-old children with conduct disorder and offering support through an evidence-based parenting programme, the invested support would yield savings of £16,400 per family over the following 25 years compared to the cost incurred if no treatment were provided (Bonin et al. 2011).

Numerous RCT’s with parents of school-aged children have shown that parenting groups are effective in improving children’s mental health and reducing their behavioural problems (Barlow et al. 2010, 2014; Hutchings et al. 2007). Recent research by King et al. (2015) demonstrated that mothers with children younger than 2 years-of-age who attended community-based parenting groups demonstrated increased maternal sensitivity and reduced maternal depression.

The evidence for positive outcomes from programmes in the first year of parenting is limited, with the majority of the research reporting on expensive, individual support. A meta-analysis by Bakermans-Kranenburg et al. (2003) included 81 intervention studies that started before the child was 54-months of age, however only 33 % of the interventions were delivered outside the home and the review did not specify which interventions were group-based. The RCT’s in their review (n = 51, including 6282 mothers) showed that interventions at this early stage of parenting had a small effect in enhancing maternal sensitivity (d = 0.33) and infant attachment security (d = 0.22).

The Nurse-Family Partnership (NFP) is an individual support programme providing weekly home visits for targeted first-time mothers from the antenatal period until the child is 2 years of age (Olds et al. 2010). Extensive research on the NFP in the USA has demonstrated long-term benefits. These include improved pre-natal mental health, longer spacing between the birth of the first and second child, reduced childhood injuries, improved school readiness and reduced dependency on welfare payments (Goodman 2006; Olds et al. 1998, 2007). A pilot trial of the programme in the UK was promising but, a recent RCT evaluation in England has failed to show short-term benefits in levels of smoking during late pregnancy, hospital admissions for children and the proportion of second pregnancies in the first 2 years (Robling et al. 2015).

Intensive home visits by nurses to low-income families from pregnancy and throughout the first 2 years of parenting cost $9600 per family (Lee et al. 2012) and in times of financial constraints; this limits the number of mothers for whom such support can be provided. Groups have been shown to be six times as cost effective as individual and clinic support (Cunningham et al. 1995).

Only a few studies have made direct comparisons of individual and group-based parenting programmes. Research with teenage mothers showed greater reductions in behaviour problems at home and better maintenance of these gains at 6-month follow-up after attending a group programme compared to individual support (Coren et al. 2003).

The Incredible Years (IY) parenting programmes are part of a suite of group-based programmes for parents, children and teachers, developed by Webster-Stratton (Pidano and Allen 2015; Webster-Stratton 2011). The IY parent programmes are based on social learning theory principles, including the modelling of positive parenting practice in groups. Parents are encouraged to establish a positive relationship with their children through joint activities and praise. Groups are delivered by two trained leaders and parents collaboratively identify core parenting principles during group discussion following viewing pre-recorded vignettes of parent–child interactions. These principles are then practised in the group and home activities are set.

The Welsh Government Parenting Action Plan (2005) supported the provision of the Incredible Years parenting programmes with resources and training for group leaders to enable them to deliver the programme to a high standard and with fidelity to the manual. This support enabled a range of Incredible Years (IY) programmes to be delivered and evaluated in real world settings. Evaluation of the IY parenting programme within Sure Start areas with parents of identified high-challenge 3- and 4-year-old children demonstrated significant improvements in child behaviour, parental mental health, positive parenting and a range of other measures (Hutchings et al. 2007) with benefits maintained at the 18 month follow-up (Bywater et al. 2009). Following this evidence of successful outcomes for the IY parenting programmes in Wales, the IY Baby and Toddler parenting programmes (Webster-Stratton 2008) were introduced to support families in the early pre-school years. The Welsh Government funding supported leader training for over 475 parenting workers to deliver these IY programmes. The 12-week toddler programme was evaluated in an RCT study which reported significant improvements in observed parental praise and improved maternal mental well-being relative to waiting list control mothers at 6 months with significant improvements at 12 months for the intervention sample only for child development, home environment, and parental depression by which time control families had been offered the intervention (Hutchings et al., submitted). Recently reported significant improvements in self-reported parenting confidence and mental well-being in parents who attended the Incredible Years Parent and Baby programme (IYPB) community groups in Wales is encouraging (Evans et al. 2015). However, the study by Evans et al. (2015) only collected pre- and post-course measures in the groups and did not have any control comparison parents.

This paper reports data from the first comparison study of the IY Parents and Babies programme in which data was collected by independent researchers. Based on the existing literature, we hypothesised that mothers attending the group would show more positive parenting behaviours than mothers in the control condition and that maternal parenting confidence and mental well-being would improve as the result of the intervention.

## Method

### Participants

**Fig. 1:**
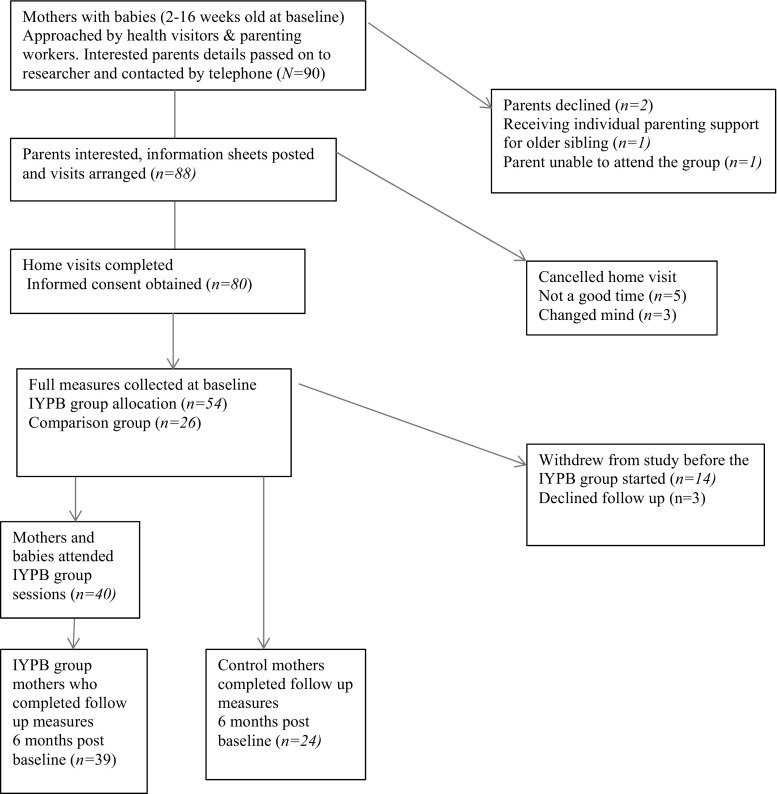
Consort diagram of the numbers of families involved in the study

The study’s eligibility criteria required that mothers had infants aged between 2 and 16 weeks at baseline and who were living in an area where trained leaders were planning to deliver the IYPB programme within the study schedule. Mothers who had previously attended an IY parent programme or were currently receiving individual parenting support were not eligible to take part in this study. Eligible mothers were invited by group leaders to take part in the study (see Fig. 1 for details), and once they indicated their willingness to participate, informed consent was obtained by the researcher (first author) at a prearranged home visit. Baseline data was also collected at the same visit and mothers were encouraged to interact with their baby in their first language.

A summary of the demographic characteristics of all mothers who provided baseline data is presented in Table 1. All study participants were biological mothers with a mean age at baseline of 26.94 years (range 17–44, SD = 5.93). The mean age of mothers leaving full-time education was 16.67 years (range 13–22, SD = 1.49). The infants were 52.4 % male and Welsh was the first language for 12 mothers, (19.0 %) equivalent to national proportions (Welsh Government 2015) with the remainder identifying English as their first language (n = 51, 81.0 %). The infants mean age at baseline was 12.5 weeks (range 3–29), with 63 dyads providing follow-up data, 6 months post-baseline (M = 26 weeks between visits, SD = 8).

**Table 1:** Demographic data collected at baseline for all recruited families

Demographic data	IYPB (*n* = 39)	Control (*n* = 24)	Attrition (*n* = 17)^a^
	Mean (SD)	Mean (SD)	Mean (SD)
Infant mean age (weeks)	12.27 (4.96)	14.71 (5.51)	8.94 (3.33)*
Mother age at first birth	22.58 (5.80)	24.33 (4.67)	22.29 (4.20)
Mother mean age	26.38 (6.23)	28.13 (5.35)	27.06 (6.36)

	Count (%)	Count (%)	Count (%)
Male index child	19 (47.5)	14 (58.3)	10 (58.8)
First born	20 (50)	13 (54.2)	8 (47.1)
Teen pregnancy	16 (17.5)	3 (12.5)	5 (29.4)
Single parent	10 (25.0)	5 (20.83)	3 (17.64)
Single/living apart	11 (27.5)	2 (8.3)	7 (41.18)
Cohabit/married	29 (72.5)	22 (91.7)	10 (58.82)
Neither working	9 (22.5)	4 (16.7)	6 (35.3)
Both in work	21 (52.5)	15 (62.5)	8 (47.06)

^a^14 did not attend any IYPB group meetings, one attended eight meetings but did not attend follow up and two were mothers from the control group

* *p* = .002

## Procedure

This was a repeated measures (pre-post) quantitative study with the parental self-report and systematic observational data collected by the researchers in the community. Mothers were invited to take part in a study and allocated on a first come first served basis to compare usual care (provided health and social care services) plus attendance at the 8 week IYPB parenting programme to usual care alone (control). The study was approved by the Welsh NHS Research Ethics Committee (10/WNo01/40) and registered with ISRCTN (number ISRCTN62055412).

Once a sufficient number of families were recruited for an IYPB group, five of the nine areas continued to recruit waiting list control families. These were areas that planned to deliver the IY toddler programme and the recruited waiting list control parents were informed they would be offered places on the IY toddler programme after the 6-month follow-up assessment. Fourteen mothers who provided baseline data and were offered a place on the IYPB programme did not attend any sessions; another parent who attended the group and two control parents declined to complete follow-up interviews (see Jones et al. 2012 for more details) this paper reports the data from the parents for whom both baseline and follow-up measures were available.

All group leaders had received the 2-day IYPB leader training and each group was co-led by two leaders (N = 17, one leader co-led two groups). The leaders were mainly health visitors (n = 10), other professions included family centre managers, specialist behaviour practitioners, parenting workers, educational and child psychologists. The majority of the leaders (n = 14, 82 %) were leading their first IYPB group and five leaders (29 %) had not delivered any IY parent programmes prior to this study. Each group leader was invited to attend weekly supervision during the intervention provided by a qualified IY mentor. The group leader supervision achieved 78 % attendance by at least one leader from each group.

The IYPB groups (N = 9) were delivered during the daytime, in community settings in nine towns in North- and Mid-Wales. Mothers attended the group with their infants for 8 weekly 2-h sessions (Webster-Stratton 2008). Group leaders recorded IYPB attendance for the parents (mean attendance 6.82 sessions, SD = 1.88 with 34 (85 %) attending six or more sessions (75 % of the programme) and receiving IY certificates for successful completion of the programme (Jones et al. 2012).

All material required for the groups, including bilingual parent handouts, agendas, stationery and small gifts for parents who completed weekly assignments were provided from the research centre. A budget of £30/parent was provided from research funds for meals/snacks for group members. Group leaders delivered the sessions according to the programme manual (Webster-Stratton 2008) and encouraged parental attendance through the core intervention strategies of weekly telephone calls and a buddy system linking group members with each other for additional support. The end of programme feedback obtained from parents (n = 34) and leaders (n = 13) was positive. Retention and attendance rates were high. A detailed costing for the groups indicated that the programme was delivered for a reasonable cost to meet health visitor objectives with families in the first postpartum months and provided health visitors with an opportunity to inform parents about resources and other support available for them and their infants (Jones et al. 2012).

The first author arranged and undertook the entire home visits to collect the data. Visits were arranged when no other visitors or distractions were present. Mothers were given £10 at each time-point as an acknowledgement of their contribution to the study. This paper reports on data collected from mothers at two-time points (at baseline recruited to IYPB group n = 54, and n = 26 control) with follow-up data (Time 2) collected 6 months post-baseline.

## Measures

### The Parent Infant Play Observation code (PIPOc) (Jones et al. 2014)

The PIPOc uses a 10-s interval partial time sampling technique to assess the six positive parent behaviours selected in line with the IYPB programme (defined in Table 2). The numbers of intervals when the behaviours were observed were totalled for the 10-min video recording of mothers’ play with their infants. No toys were allowed in the first 5 min and a standard toy was introduced for the subsequent 5 min. Parent behaviours were coded independently by a graduate who was trained by the first author using the PIPOc manual (Jones 2013). The coder remained blind to the group allocation of observed parents. Factor analysis of the data resulted in the six positive parenting behaviours forming three main behaviour components (Sensitive Parenting, Physical Encouragement and Verbal Engagement). Global scores for the PIPOc were produced by summing the values for these three positive parenting behaviour components. The PIPOc has shown strong to excellent intra-class correlation (ICC) test–retest reliability scores on each target behaviour (N = 6) observed over a 17-day interval (n = 15) ICC single measure = .7–.9. Inter-rater agreement was also strong to excellent (n = 37) ICC single = .7–.9. Finally, preliminary tests of concurrent validity between the PIPOc and subscales of the Infant–Toddler Home Observation for Measurement of the Environment inventory (IT HOME, Caldwell and Bradley 2003), resulted in correlations at both time points (Jones et al. 2014).

**Table 2:** PIPOc behaviours

Coded behaviour	Definition	PIPOc components
Talk	Any neutral or positive vocal cues from the parents who encourage their infants to recognise sounds and label objects in their environment	Verbal engagement
Touch	The parent physically touches or holds their infant in a warm affectionate manner	
Play	Parent proactively initiates and sustains games with their infant with obvious positive affect as the parents’ attempts to engage their infants’ interest	Physical encouragement
Move	The parents encourage their infant’s fine and gross motor movement, promoting the infant’s physical development	
Mind	Parents verbalise the child’s wants or emotions and help them label, identify and understand their emotions	Sensitive parenting
Respond	Parents respond in a neutral or positive manner to their child’s neutral or positive vocal or physical action	

### The Karitane Parental Confidence scale (KPCS; Črnčec et al. 2008a, b)

This parental self-report measure was developed to assess parenting confidence for parents of children aged 0–12 months. Parents select their most appropriate answer from fifteen items rated on a Likert scale that is scored 0, 1, 2 or 3 with a total score range of 0–45. Higher scores indicate higher confidence as a parent. The scale has been validated (N = 187 mothers) and convergent validity was established with correlations between the total score and four dependant measures assessing parenting competence, confidence, depression and stress (Črnčec et al. 2008b). The KPCS total score internal consistency score using Cronbach’s alpha at ∝ = .81 is above the recommended .70. Test–retest reliability assessed 4 weeks after initial administration resulted in r(26) = .88. Kohlhoff and Barnett (2013) report the KPCS total mean scores from mothers (n = 83) with infants mean age of 5.3 months (SD = 3.2) enrolling on a 4-day residential programme to resolve parenting problems to be 33.70 (SD = 5.92).

The Warwick-Edinburgh Mental Well-being Scale (WEMWBS; Tennant et al. 2007) was developed to assess the population mental well-being of adults in the UK. The scale includes hedonic and eudemonic aspects of mental health including positive affect (feelings of optimism, cheerfulness, and relaxation), satisfying interpersonal relationships and positive functioning (energy, clear thinking, self-acceptance, personal development, competence and autonomy). This self-report measure includes 14 items answered using a 1–5 Likert scale with a total score range of 14–70. The scale developers state that general population internal consistency tests resulted in Cronbach’s alpha of ∝ = .91. Test–retest reliability after 1 week between completions was also very good at ∝ = .83 (Stewart-Brown and Janohamed 2008). Tennant et al. (2007) reported the population mean score to be 50.7 with a 95 % confidence interval (50.3–51.1). The WEMWBS has been shown to be responsive to change following intervention at the individual and group level (Maheswaran et al. 2012).

The Infant–Toddler Home Observation for Measurement of the Environment inventory (IT HOME, Bradley and Caldwell 1976; Caldwell and Bradley 2003) is based on the ecological model of development (Bronfenbrenner and Morris 1998). The IT HOME inventory includes 45 binary scored items that evaluate the provision of resources and nurturing activities within the home. The researcher observed or clarified with the parent during the home visit whether the infant engaged in various age appropriate activities. Evaluations of the HOME have shown excellent inter-rater reliability (>90 %) (Saudino and Plomin 1997). Moderate stability for the total HOME scores (with Cronbach’s alpha = .77) have been reported with 12–24 month old infants from low-income families (Shaw and Vondra 1995). A review of the measure by Totskia and Sylva (2004) found mothers who exhibited more sensitivity and responsiveness on the HOME inventory were more likely to have securely attached children when assessed at 36-months of age (according to the MacArthur system of attachment).

The Griffiths Mental Development 0–2 year Scales, (GMDS; Griffiths 1954; revised Huntley 1996), was validated using a British sample (N = 571) (Griffiths 1954) and later revised by Huntley (1996) (N = 665). A standardised set of items were used during the home visit by the trained researcher to test the infant’s developmental profile on five subscales: Locomotor, Personal-Social, Language, Hand and Eye coordination and Performance. The total scores are also used to calculate the child’s age equivalent and ‘general quotient’ (GQ).

Data on the demographic circumstances, health and social information of the parents and children were obtained using a revised semi-structured interview based on the Personal Data and Health Questionnaire (PDHQ; Hutchings 1996) with additional items related to the first postpartum year included for this study (Jones 2013).

### Data Analyses

Independent samples *t*-tests and Pearson’s Chi-Square tests run in crosstabs were performed to compare the baseline data collected from parents who remained in the study at Time 2 (N = 63) and those who withdrew after baseline measures were collected (n = 17) to identify any differences on the demographic variables at baseline. The analysis is based on participants who provided full data sets at both time points (N = 63). The relation between baseline and follow-up measures were assessed for the whole sample by using Pearson product moment correlations. Notable findings are reported; a full breakdown of the correlational analysis is available upon request. To identify whether there were any significant changes across the whole sample over time, paired samples *t*-tests were run on the main target variables.

Due to the exploratory nature of the study within-group, pretest-postest analyses of the main measures were undertaken with paired samples *t*-tests to assess whether there was a significant change over time within each group. To reduce Family Wise and Type 1 errors tests were run on the global scores for each measure independently. The distributions of the main outcomes, Skew and Kurtosis values for each outcome variable at baseline and follow-up are reported in Table 3.

**Table 3:** Overall mean scores at baseline and follow up. (N = 63)

Variable	Baseline			Follow up			*t*	*p*	95 % CI	*d*
	Mean (SD)	Skewness (SE)	Kurtosis (SE)	Mean (SD)	Skewness (SE)	Kurtosis (SE)				
KPCS	40.87 (3.29)			41.54 (3.14)			1.71	.094	−1.45 to .117	0.21
WEMWBS	52.95 (7.82)	−.42 (.30)	.46 (.60)	52.49 (8.39)	.30 (.30)	−.06 (.60)	0.49	.627	−1.43 to 2.35	−0.06
Griffiths GQ	101.94 (10.64)	−.06 (.30)	.12 (.30)	109.74 (11.35)	.12 (.30)	−.25 (.60)	4.42	.001**	−11.34 to −4.27	0.71
IT home	23.27 (3.82)	−.70 (.30)	−.40 (.60)	27.03 (3.34)	−.70 (.30)	−.35 (.60)	7.61	.001**	−4.73 to −2.76	1.05
PIPOc global	99.84 (26.04)	.27 (.30)	−.50 (.60)	111.50 (22.81)	−.47 (.30)	.18 (.60)	4.12	.001**	−20.75 to −7.19	0.51
PIPOc physical encouragement	40.31 (15.31)	.04 (.30)	−.53 (.60)	39.06 (12.18)	−.30 (.30)	.04 (.60)	0.17	.869	−4.47 to 5.25	−0.03
PIPOc verbal engagement	41.89 (14.47)	.20 (.30)	.35 (.60)	55.85 (16.26)	−.42 (.30)	−.70 (.60)	5.95	.001**	−20.63 to −10.25	0.87
PIPOc sensitive parenting	17.65 (10.04)	.66 (.30)	−.33 (.60)	16.58 (9.42)	1.11 (.30)	1.34 (.60)	0.63	.530	−2.31 to 4.43	−0.11

Finally, baseline PIPOc sub-variable scores were included as covariates in three ANCOVA models run on the three PIPOc components; this allowed for any treatment related changes in follow-up scores that were due to the treatment to be elucidated. The dummy coded dichotomous treatment condition variable was included in all models as a fixed factor.

## Results

Analysis showed that parents who were allocated to the intervention and control conditions were well matched; there were no significant differences between parental age, infant age, infant gender, target child’s birth order position in the family, proportion of single parents, young mothers, PIPOc and IT HOME scores, parenting confidence (KPCS), mental well-being (WEMWBS) or infants’ GMDS GQ scores at baseline.

Infant’s age at baseline was the only significant difference between families who chose not to progress after baseline measures were collected and those that remained in the study. The families who left the study had younger infants (M = 8.94 weeks, SD = 3.33) than those who remained in the study (M = 13.27 weeks, SD = 5.26), F (78.1) = 1.672, *p* = .002. Figure 1 shows a consort diagram of the numbers of families who were interested and involved in the study.

Parental self-reported mental well-being and confidence at baseline were high in both groups, suggesting that the mothers were generally functioning well. The WEMWBS mean score of 52.95 (SD = 7.82) was higher than the population mean of 50.7 (Tennant et al. 2007). The KPCS baseline mean score was also found to be higher (40.87, SD = 3.29) in comparison to the data reported by Kohlhoff and Barnett (2013); 33.70 and SD = 5.92, again suggesting this was a well-functioning sample at baseline.

Significant increases in Griffiths GQ, IT HOME, PIPOc Global and PIPOc Verbal Engagement component scores were identified from baseline to follow-up across the whole sample in this study. However, no significant changes were identified in KPCS, WEMWBS and PIPOc Physical Encouragement and PIPOc Sensitive Parenting scores across the whole sample over time. The distributions of the main outcome variables are reported in Table 3.

A small kurtosis violation of normality values at follow-up was observed on the PIPOc Sensitive Parenting component; however this did not have a major effect on the direction of the results. These scores are a reflection on the observed behaviours coded independently from the videos. Changes over time within both groups were investigated using the reported *t*-tests because a repeated or mixed measures ANOVA was considered to be too insensitive to detect the behavioural changes between groups in this sample, because of the pre-post changes in target variable scores within groups. The differences on the target variable scores from baseline to follow-up for each group are presented and summarised in Table 4.

**Table 4:** Within-group differences for main target variables

Variable	IYPB (n = 39)					
	Pre mean (SD)	Post mean (SD)	*T*	*p*	95 % CI	*d*
KPCS	40.95 (3.41)	41.38 (3.38)	0.78	.439	−1.56 to 0.69	0.13
WEMWBS	55.00 (7.61)	53.18 (8.66)	1.46	.154	−0.71 to 4.35	−0.22
Griffiths GQ	102.03 (10.70)	110.76 (11.12)	3.98	.001**	−13.18 to −4.24	0.77
IT home	22.15 (3.81)	27.03 (3.52)	7.63	.001**	−6.17 to −3.58	1.33
PIPOc global	95.61 (26.50)	111.29 (32.94)	3.46	.001**	−24.88 to −6.49	0.53
PIPOc physical	39.13 (14.98)	37.53 (14.25)	0.51	.612	−4.71 to 7.97	−0.11
PIPOc verbal	41.11 (16.52)	55.24 (23.69)	3.98	.001**	−21.33 to −6.93	0.70
PIPOc sensitive	15.37 (9.69)	18.53 (10.33)	1.57	.125	−7.23 to 0.91	0.32

Variable	Control (n = 24)					
	Pre mean (SD)	Post mean (SD)	*T*	*p*	95 % CI	*d*
KPCS	40.75 (3.14)	41.79 (2.73)	2.11	.046*	−2.07 to −.019	0.35
WEMWBS	49.63 (7.13)	51.38 (7.99)	1.32	.199	−4.49 to 0.99	0.23
Griffiths GQ	101.79 (10.77)	108.13 (11.79)	2.11	.046*	−12.54 to −.124	0.56
IT home	25.13 (3.11)	27.04 (3.09)	3.09	.005**	−3.19 to −.635	0.35
PIPOc global	106.54 (24.33)	117.79 (21.24)	2.22	.037*	−21.74 to −.754	0.49
PIPOc physical	42.17 (15.95)	43.67 (12.56)	0.38	.705	9.60 to 6.60	0.32
PIPOc verbal	43.13 (10.66)	60.63 (15.70)	4.74	.001**	−25.14 to −9.86	1.33
PIPOc sensitive	21.25 (9.72)	13.50 (6.90)	3.17	.004**	2.68 to 12.81	−0.93

*,** Significant change in scores from baseline (pre) to follow-up (post)

Both groups showed significant increases in mean Griffiths GQ scores from baseline to follow-up. These results may reflect improved developmental outcome or more accurate assessment as the infant develops more items were available for assessment. Significant improvements were also shown in the IT HOME scores of both the intervention and control groups over time, with the intervention group showing a very large effect size.

Neither the control nor IYPB group showed any significant changes in WEMWBS scores from baseline to follow-up. This may be because both groups demonstrated high baseline scores on this measure, other studies with at-risk families referred for interventions reported lower baseline values of 42.6 (SD = 9.4) (Family Links; Grant 2012), 42.9 (SD = 10.3) (PEIP, Lindsay et al. 2011) and 43.37 (SD = 10.18) (IY Toddler evaluation; Griffith 2011).

The control group reported a (0.12) increase in mean KPCS score from baseline to follow-up, which was significant (*p* = .046). A smaller, non-significant, increase was also evident in the KPCS scores of the IYPB group scores over time. Both groups reported mean baseline scores which were higher than the post group data reported by Evans et al. (2015) and Črnčec et al. (2008a, b).

The results from the PIPOc observational measure comparing baseline and 6-month follow-up data showed both groups increased in Global positive parenting and PIPOc Verbal Engagement. Neither group showed any significant changes in PIPOc Physical Encouragement scores from baseline to follow-up.

The IYPB group showed no significant changes in PIPOc Sensitive Parenting scores from baseline to follow-up. However, the control group were observed to show a significant reduction (*p* = .004) in PIPOc Sensitive scores from baseline to follow-up.

Variables such as mothers’ age at first birth, parity, marital status and baby’s gender were excluded as covariates from the ANCOVA analyses because they were found to have no overall effect on the direction of the results. The first two models showed no main effect for treatment condition on PIPOc Physical Encouragement and Verbal Engagement components follow-up scores after controlling for baseline scores with both subscales showing an overall improvement for both groups. However, the final model for the PIPOc Sensitive Parenting sub-variable did show a main effect for treatment condition after controlling for the baseline covariate score, *F*(1,59) = 5.66, *p* = .021, partial eta-squared = .088. The IYPB group (*M* = 18.87, *SE* = 1.51; 95 % CI 15.86–21.88) were found to score significantly higher at follow-up than the control group (*M* = 12.96, *SE* = 1.91; 95 % CI 9.13–16.79), a mean difference of 5.91 (*SE* = 2.48; 95 % CI 9.42–10.88). The IYPB intervention appears to have been effective in increasing mothers’ Sensitive Parenting scores relative to the changes in control parents observed sensitivity.

## Discussion

The caregivers in this study were all biological mothers, Caucasian and living in rural areas of Wales. Therefore, the results cannot be generalised without replication in urban or multi-cultural samples, although other IY programmes have been shown to be equally effective with parents from different cultural backgrounds (Reid et al. 2003). At present, we do not know whether the IYPB intervention would be effective with fathers or other carers.

Despite the practical and financial constraints within the present research, which prevented an RCT design, baseline data on key demographic and outcome measures showed that intervention and waiting list control group parents were well matched. The groups were all delivered within a schedule that enabled leaders to join other IYPB group leaders in weekly supervision. This support was valued by the leaders, as many were delivering their first IYPB groups; however, the schedule for the groups and relatively low birth rates within the recruitment areas limited the number of families who were eligible to take part.

The requirement for parents to opt in/self-select to the study appears to have resulted in parents with above average levels of mental well-being and confidence taking part, possibly reducing the scope for improvement in these areas following a brief intervention. The group means for KPCS in this study were higher than the mean scores reported by the scale developers (Črnčec et al. 2008a, b; Kohlhoff and Barnett 2013) and parents in a targeted recruitment to IYPB groups over a longer period reported by Evans et al. (2015) demonstrated lower self-assessed parenting confidence and mental health at the start of the programme. Essentially, the parenting confidence of the study sample was high at the outset, which limited the potential for improvement. Similarly, baseline mental well-being scores (WEMWBS) were also higher in this study than in twelve other reported studies that have previously utilised this measure during intervention studies (Maheswaran et al. 2012). The high group baseline mean scores for parent-reported mental well-being and confidence suggest that the opt-in mechanism for recruiting families to the evaluation may have attracted mothers who were motivated and self-assured in relation to their skills as a parent, leaving less scope for improvement after an 8-week programme.

The parents in both intervention and control conditions within our study showed improvements on the infants Griffiths GQ scores, IT HOME ratings, and parents observed PIPOc Global and PIPOc Verbal Engagement scores. The universal access to services as usual which in the UK are well developed (Hall and Elliman 2006) may have had a positive impact on the families irrespective of their status in this study. A larger study which monitored use of other services would be justified to increase our understanding of the benefits gained from parenting support.

Research by Hutchings et al. (2007) evaluating the preschool IY programme included a screening tool for parents recruited to the study, hence ensuring the programme was delivered to parents who were most in need of additional support. Future research should consider targeting recruitment of parents with infants between 3 and 6 months of age at baseline who may be at higher risk of poorer outcomes. The significant benefit for the intervention group in observed maternal sensitivity was not evident in the rating of maternal warmth by the researcher using the IT-HOME subscale. Although we found the expected associations between the PIPOc global scores and IT- HOME measure, the latter measure was possibly less sensitive to change in the mothers’ interactions with their infants. This finding is consistent with the literature that promotes the use of direct observational measures over indirect assessments of behaviour (Aspland and Gardner 2003), and provides another justification for independent observation.

The observed positive parenting behaviours: Verbal Engagement and Global PIPOc scores, increased for the whole sample with no significant differences observed between the two groups. It is possible that in the early months of the infants’ lives, mothers adapted their behaviours as the infants became more alert and that this is a natural change in mother-baby interactions as shown by the longitudinal analysis of maternal responsiveness by Bornstein et al. (2008). Further research with a larger sample would be needed to confirm this shift in parenting behaviour to coincide with infant development. The encouraging benefit of intervention on PIPOc Sensitive Parenting in this study may facilitate infants secure attachment, an important protective factor in children at increased risk of poorer outcomes.

The IY programmes have fidelity tools that include basic leader training; manual and other resources for parents including the IY baby book which was published after this study. In addition, there is a rigorous leader certification process as part of which leaders submit a tape of a full session of delivery of the programme and this is rated for the key leader collaborative process skills. This is a quality control process that provides evidence that the programme is being delivered as intended. Most of the mothers in the intervention group had group leaders who were delivering the programme for the first time and none had achieved leader certification so it is likely that the present results underestimate the true effectiveness of the IYPB intervention. A trial with certified leaders who have more experience in programme delivery is needed to establish if experience and certification of leaders results in better outcomes.

The IYPB group leaders encouraged mothers to observe their infants and the video vignettes modelled how to respond appropriately to their infants’ cues. We predicted that mothers attending this group-based intervention would interact more positively and encourage their infants’ development. It seems likely that this was effective, as we found that the IYPB intervention increased mothers’ Sensitive Parenting PIPOc scores relative to the control group. The size of this improvement in maternal sensitivity in the group of parents who attended the IYPB (Cohen’s d = 0.32) was comparable to the effect sizes reported in Bakermans-Kranenburg et al. (2003) review of RCT intervention effects on maternal sensitivity (d = 0.33).

The results from this study have higher ecological validity than laboratory-based research since all the data including the observations of mothers playing with their infants were collected in the home. The results of the IYPB intervention evaluated in the present paper suggest that although the parents were already functioning well at the start of the programme, it successfully increased the mothers’ sensitivity to their infants’ needs relative to control parents over the same time period.

Although the demographic and health data collected in this study showed parents were well matched, the small sample size limited the potential for any moderator and mediator analyses of the results. The parents and infants in the study had access to other support programmes in their community and this reflects the challenges of evaluating such interventions within communities already receiving universal early years support. The other challenge involved evaluating a brief intervention targeted at promoting positive parenting and preventing problems from developing when only short-term outcome measures were available.

The IYPB programme has the potential to impact on attachment and support the establishment of stable relationships at a crucial period of development. A larger RCT delivered by experienced and certified IYPB leaders working with parents at greater risk for poorer outcomes with service use followed from birth into school-age would be justified. This would further increase our understanding of the value of primary preventative parenting interventions delivered to families in the first year of their child’s life.
